# Novels as data: Health humanities and health psychology

**DOI:** 10.1177/1359105321999107

**Published:** 2021-03-11

**Authors:** Ad A Kaptein

**Affiliations:** Medical Psychology, The Netherlands

**Keywords:** art, health humanities, health psychology, interpretative phenomenological analysis, medical humanities, novels

## Abstract

Novels represent a corpus of data that offers innovative opportunities for research and theory in health psychology. This article discusses how adding ‘health humanities’ to health psychology opens up a potentially rich domain for research and clinical application. The concept of ‘health humanities’ is discussed and put into a context of related fields. The concepts of ‘illness perceptions’ and ‘models of patient–health care provider interaction’ are used as illustrations. Applications are given, focusing on patients and their caregivers, health care providers and society at large (bibliotherapy and expressive writing). Suggestions for further development of the area are included.

“ . . . the novel is sogged with humanity . . . ” E.M. Forster (1927)

Health psychology is a relatively young area of scientific endeavor which enjoys a quite astonishing growth over the past decades (Quinn et al., 2020). Journals, Societies, conferences, university departments and research output attest to this development, illustrating the scientific and societal relevance of health psychology as a scientific discipline. Research methods in health psychology are to a great extent comparable to those in other areas of psychology. In addition to questionnaires, interviews, observational techniques, archival data, methods that focus on more physiological phenomena and concepts are fairly specific and typical of methods in health psychology (e.g. assessing blood pressure, HbA1c, pulmonary function, using MRI, etc.). Given the proximity of health psychology to the world of medicine, health psychology borders on fields such as behavioural medicine, medical anthropology and social medicine, and also on fields focusing on ‘patients’ stories’, such as literary studies, medical humanities and psychiatry (Cole et al., 2015; Crawford et al., 2015; Jones et al., 2014). ‘Medical Humanities’ seems to be the term that is used to indicate the area of research and clinical care in the broad domains of humanities, social science and art. The definition of Medical Humanities by Aull (2006) and Brody (2011) illustrates the point: ‘an interdisciplinary field of humanities (literature, philosophy, ethics, history and religion), social science (anthropology, cultural studies, psychology, sociology) and the arts (literature, theater, film and visual arts) and their application to medical education and practice’. Medical Humanities represents a more or less established area as illustrated by scientific journals such as *Journal of Medical Humanities*, *Medical Humanities* and by medical humanities niches in established major medical journals (e.g. *JAMA*, *Lancet* and *New England Journal of Medicine*).

Health psychologists will note the biomedical perspective in the definition of Medical Humanities: ‘. . . and their application to medical education and practice’. The rather recent introduction of the concept ‘Health Humanities’ reflects the growing impact of health psychology and related health and social sciences in this area: health humanities attempts to adopt a biopsychosocial view rather than a biomedical view, emphasizing the patient’s position, the experience of being ill and of living with a chronic illness; furthermore, health humanities incorporates non-medical professionals in its remit (i.e. caregivers, patient representatives, (health) psychologists, etc.). The advent of the biopsychosocial model which helped the development and growth of health psychology seems also discernable in the shift from Medical Humanities to Health Humanities: ‘. . . in fact, the terminological shift from *medical* to *health* humanities underscores the crucial distinction between medicine and health’ (Jones et al., 2017, p. 933). As pointed out by Jones and colleagues, developing new labels for a scientific discipline is more than word play. Health Humanities is defined as ‘the interdisciplinary field of study that draws on aspects of the arts and humanities in its approach to health care, health and well-being . . . the application of the creative or fine arts (including visual arts, music, performance arts) and humanities disciplines (including literary studies, languages, law, history, philosophy, religion, etc.) to questions of human health and well-being’ (Crawford et al., 2020).

One of the ‘founding fathers’ of Health Humanities outlines a set of core characteristics of the concept. According to Crawford, Health Humanities focuses on all the professional personnel involved in health care, health and well-being, as well as on informal or unpaid carers and the self-caring public, applying arts and humanities to the benefit of a society’s health care and social well-being, democratizing therapeutic interventions beyond specific professionals and enhancing health care environments (Crawford, 2015, p. 2). These characteristics will sound familiar to health psychologists aware of Ivan Illich’s work (Illich, 1976) where he maintains how medical health care may be a danger to an individual’s and society’s health. Illich’s views are reverberated in the statement in the introductory chapter of the Health Humanities Reader: ‘. . . overwhelming evidence suggests that health and its distribution in human populations is mostly not the result of medical care (which is not to deny the myriad ways in which the latter is both meaningful and important) . . . any definition of the health humanities cannot be limited to the field of medicine, medical care or medical professionals, and it also should not have as its central goal the advance of the practice and science of medicine . . . our primary goal should be directed to health and human flourishing rather than to the delivery of medical care; the two objectives are actually not nearly so tightly interwoven as most tend to think, even if both of them are independently of great worth’ (Goldberg, in Jones et al., 2014, p. 7). The emphasis within the Health Humanities domain on well-being rather than on the absence of disease, on all the participants in society rather than on health care providers, on social as well as individual determinants of health, reflects the points raised by Spicer and Chamberlain in their paper ‘Developing psychosocial theory in health psychology’ (1996) in this Journal.

Giving meaning to symptoms and illness is a core subject in psychology as applied to health and medicine. Weinman delineates three areas of research and clinical intervention as major areas of health psychology: health behaviour, illness behaviour and patient-health care provider interaction (Weinman, 1981). The last two areas in particular are relevant in the context of this article. Biomedical views on how people make sense of illness and respond to illness were (and still are) often labelled ‘lay representations of illness’, with the associated connotation of ‘lay’ as ‘ignorant’, ‘irrelevant’, ‘inferior’. Mainstream psychology offered ‘attribution theory’ (Heider, 1958) and ‘folk psychology’ (Bruner, 1990) as approaches to studying representations of illness by non-medical persons, that is, those without a medical gaze or biomedical model. Various theoreticians from outside the medical domain question the strict biomedical model. Cultural anthropologist and psychiatrist Kleinman (1988) introduced the concept of ‘explanatory model’ for describing the sense making by ‘lay persons’ of symptoms and signs, emphasizing and acknowledging the importance and relevance of conceptions by ‘lay’ persons of their symptoms and sense making. Sociologist Arthur Frank contributes to the area of ‘making sense of illness’ in a number of books that focus on how ‘illness narratives’ drive patients’ illness behaviour. His three categories of illness narrative, ‘restitution’, ‘chaos’ and ‘quest’ narrative, offer health psychologists rich research opportunities when studying the oral or written accounts of ‘being ill’ by ‘the wounded storyteller’ (Frank, 1995). In health psychology, the work by Howard Leventhal is a major contribution to the area of how people make sense of symptoms, signs and illness (Leventhal et al., 2003). His Common Sense Model is one of the leading theoretical models in health psychology, with solid research and sound methods of assessment of illness perceptions (questionnaires) and additional approaches (drawings of illness, graphic medicine), and a body of knowledge on intervening in illness perceptions in order to impact outcomes. Illness narratives are a central issue in the writing by Michael Murray, health psychologist and social psychologist. He maintains that illness narratives allow patients to ‘. . . use and create stories not only to describe and understand events but to define ourselves and others’ (Murray, 1997, p. 10). The themes that Murray discusses fit in with how individuals, healthy or ill and the social systems they live in produce ‘images of illness’ that shape how ill persons attribute meaning to their illness, with its consequent impact on health care seeking behaviour, illness behaviour and self-management (Murray, 2000). These themes, of course, make up major building blocks of illness narratives in novels as well. Various Special Issues on the subject of illness narratives, writing and their position regarding theory and methodology in health psychology have been published (e.g. Murray, 2009; Murray and Gray, 2008; Sools et al., 2015; see also Bleakley (2005) for a discussion of ‘narrative’ as data source and unit of analysis, Chamberlain (2000) for discussion of conceptual issues and Stephens (2011) for discussions on data analysis of narratives).

Studying novels as a source of data for research in health psychology is a fairly unexplored area. One can discern a number of approaches in this area, ranging from the somewhat impressionistic style adopted in the literary sciences, to attempts at a more formal, theory-based approach, as in interpretative phenomenological analysis, for instance (Smith et al., 2009). The most basic level of using novels as data seems to be one-page papers in biomedical journals where a novel is described and used to outline the relevance of the novel for clinical work by clinicians (e.g. the article in the British Journal of Psychiatry by Wilkinson, 2019, about melancholy in Hamlet). Case histories of famous authors, their medical history and discussing (or speculating) how these histories impacted their novels (e.g. Wilson, 2012, on the eye pathology of Jane Austen) make up a second approach. Third, the representation of a specific illness in a novel, as outlined by medical authors who debate the precise diagnosis and therapeutic approach (e.g. Zayas et al., 2006), on migraine in Bulgakov’s The master and Margarita) is quite popular in medical journals. Attempts to review how a particular illness is represented in (high) literature, for example, epilepsy (Wolf, 1999) or cancer (Kaptein, 2021) are more ambitious. Finally, comparing the representations of illness in novels with empirical work on patients’ representations of the particular illness combines the literary representation with the empirical image of an illness (e.g. Broyard’s illness narrative of his prostate cancer in the context of reviews of empirical studies on the topic (e.g. Florijn et al., 2019).

## Locating relevant sources

Identifying novels is a somewhat complex undertaking given the relative limited number of relevant data-bases on the subject. Various organizations with their databases provide search systems for novels where being ill is a major theme. The Division of Medical Humanities at NYU Langone Health features the LITMED Literature Arts Medicine Database, with listings and analyses of novels, films and other art genres; a weekly Newsletter updates the search systems [www.med.nyu.edu]. The weekly Newsletter from Columbia University, Department of Medical Humanities and Ethics [narrativemedicine@cumc.columbia.edu; www.narrativemedicine.org] also provides the reader with updates on research and clinical work in various art genres in the Health Humanities area. PubMed is a useful for papers on health humanities [www.pubmed.gov] as is World Cat/Catalogue [www.worldcat.org].

Two categories of journals are solid sources of information on novels about illness. Journals specifically focusing on health humanities: *Academic Medicine; Hektoen International; Journal of Medical Humanities; Journal of Medicine and Philosophy; Journal of Writing Research; Literature and Medicine; Medical Humanities; Philosophy, Ethics, and Humanities in Medicine*. Second, journals covering the wider area of arts in medicine: *Anthropology & Medicine; Art Therapy; Arts & Health; International Journal of Art Therapy; Journal of Visual Communication in Medicine.* An impressive number of high impact factor biomedical journals have a Health Humanities niche, variously labelled as ‘pectoriloquy’, ‘humanities’, ‘literature and arts’, ‘art in medicine’, ‘Patients’ (sic), ‘when the tumor is not the target’, and ‘culture’: *American Journal of Psychiatry; Annals of Internal Medicine –* with an ‘Annals Graphic Medicine’ category*-; BMJ; British Journal of Psychiatry; Chest; Canadian Medical Association Journal CMAJ; Family Medicine; JAMA; Journal of Clinical Oncology; Lancet; New England Journal of Medicine.*

IGEL is an international society aiming at studying literature empirically, with some attention to health humanities [www.sites.google.com/igelassoc.org]; their journal [*Scientific Study of Literature*] may be useful. *The Routledge Companion to Health Humanities* offers some search strategies for identifying novels, and music, film and paintings in relation to being ill (Crawford et al., 2020), as does the Health Humanities Reader (Jones et al., 2014). Excellent material is available in the books by Engelhardt (2018) who managed to collect and systemize hundreds of novels in many languages where ‘being ill’ is a leading theme. Hoerni lists novels on cancer in the French language (Hoerni, 2016).

## Novels, health humanities, health psychology

Novels wherein two major concepts in health psychology – ‘making sense of illness and illness behaviour’ and ‘patient–health care provider interaction’ – are featured extensively, are briefly presented and discussed. ‘Nemesis’ by Philip Roth, ‘The Breath’ by Thomas Bernhard, ‘From the journal of a leper’ by John Updike, and ‘Wit’ by Margaret Edson are examples of novels where illness and the responses to illness by patients, caregivers and health care providers play a central role. *Nemesis* is a novel on an infectious disease, written by an American author. *The Breath* focuses on a pulmonary condition, written in the format of an illness narrative by an Austrian author. *From the journal of a leper* is an illness narrative on a chronic skin disorder, written by an American author. *Wit* is the Pulitzer prize winning novel/play about coping with ovarian cancer and the associated struggle between patient and health care providers. The author worked as a research nurse at a cancer institute at the time of writing her book.

‘*Nemesis*’ by Roth (2010) has as its focus the poliomyelitis (‘polio’) epidemic in the northeastern United States in 1944. At a descriptive level the novel deals with how the various groups in the population of Newark respond to the threat of the infectious disease, their causal attributions (‘the Jews!’, ‘the Italians!’, ‘the crazies!’, etc.), their preventive behaviour (‘avoid milk’, ‘wash your hands’) and their grief over young children falling victim to polio. On a more abstract level the novel is about guilt, pride and love. At another abstract level, Nemesis is part of an extensive list of novels about infectious disease in the world literature (e.g. cholera, diphtheria, HIV/AIDS, leprosy, malaria, plague, tuberculosis – e.g. Kaptein et al., 2020b), offering a myriad of options for health psychology research.

Using the Common Sense Model and its central theoretical concept of illness perceptions, operationalized in the dimensions of the Illness Perception Questionnaire (the Revised and the Brief versions), *Nemesis* offers observations that illustrate the various dimensions of illness perceptions. For instance:

Identity: ‘. . . if a child exhibited symptoms such as headache, sore throat, nausea, stiff neck, joint pain or fever . . . a paralytic disease that left a youngster permanently disabled and deformed or unable to breathe outside a cylindrical metal respirator tank known as an iron lung – or that could lead from paralysis of the respiratory muscles to death. . .’ Causes: ‘. . . they should inspect the milk that kids drink – polio comes from dirty cows and their infected milk . . . they don’t sterilize those bottles right . . . why don’t they fumigate?’, . . . Treatment control: ‘. . . try to wash yourself thoroughly every day and eat right and to get eight hours of sleep and to drink eight glasses of water a day and not to give in to your worries and fears’. Coherence: ‘polio is polio – nobody knows how it spreads . . . You can’t wash the polio away. You can’t see it. It gets in the air and you open your mouth and breathe it in and next thing you got the polio’.

The theme ‘interaction between patient and health care provider’ is illustrated with quotes from ‘*The Breath*’ by Thomas Bernhard (1981), ‘*From the journal of a leper*’ by John Updike (1978), and especially ‘*Wit*’ by Margaret Edson (1993). The theoretical model used is the paper by Emanuel and Emanuel (1992) on ‘Four models of the physician-patient relationship’. *The Breath* is the ultimate illustration of ‘the paternalistic model’: the doctor determines what the patient needs, is dominant and authoritative. The other three models (informative model, interpretative model, deliberative model) are simply absent in *The Breath*: ‘ . . . but it was impossible to speak to them . . . The doctors on the ward-round never did anything to enlighten their patients in the death ward, and in consequence all three patients were effectively abandoned, both medically and morally’ (p. 241), ‘Every day they appeared in front of my bed, a white wall of unconcern in which no trace of humanity was discernable’ (p. 240; see Kaptein and Lyons, 2009, in this Journal, for a detailed analysis).

In *From the journal of a leper* John Updike’s relation with his physician is radically different from that of Thomas Bernhard’s. ‘The doctor whistles when I take off my clothes. “Quite a case”. . . . He explains the treatment. . . . we have this type of light now. “When you clear,” he says casually, towards the end. When I clear! The concept is staggering. I want to swoon, I want to embrace him, as one embraces, in primitive societies, a madman’ (Updike, 1978, pp. 4–5).

A more formal, theory-based approach to analysing a novel, which captures the core concepts of ‘sense making’ and ‘studying responses to major life events, such as illness’ to a great extent is *interpretative phenomenological analysis (IPA)*. ‘IPA is a qualitative research approach committed to the examination of how people make sense of their major life experiences. IPA is phenomenological in that it is concerned with exploring experience in its own terms’ (p. 1, Smith et al., 2009). A critical evaluation of the use of IPA in health psychology was given by Brocki and Wearden (2006) who identified 52 studies with IPA as the theoretical approach. More recent studies in a health psychology context are, for example, Spiers et al. (2016) on the treatment experiences of people living with ileostomies, and Shahmoon et al. (2019), about people making sense of Parkinson’s disease.

Applying IPA theory and methodology was attempted in an Honours Class ‘Literature & Medicine’ for medical students (see Kaptein et al., 2012) for the outline of the class, the novels used and the publications by the participating students). The students were instructed to (a) study the somatic basis of a disease, (b) study a novel where that particular disease (the illness!) has a prominent position, (c) explore the lived experience of the illness as told by a patient with that particular illness and (d) write a paper that combines these three components. *Wit* turns out to be an extremely useful novel for this purpose as it clearly demonstrates the clash between the biomedical and the biopsychosocial approach to disease and illness (Edson, 1993). In the *Journal of Cancer Education*, we presented the results of an analysis of this novel with a focus on how a patient with cancer interacts with her oncologists, physicians with extreme biomedical views on medicine (Kaptein and Lyons, 2010; van Duin and Kaptein, 2013). Rereading *Wit* and studying ‘A study guide for Margaret Edson’s ‘Wit’ (Gale, 2001), it becomes clear that patient–health care provider interaction is a – if not *the* – major subject of the book. Rereading the novel and the related educational material (Gale, 2001) with IPA as a guide allowed identifying patient-physician interaction as the central theme of the book. The senior medical oncologist tells Vivian, the protagonist, she has stage IV ovarian cancer. She agrees to undergo an aggressive treatment of chemotherapy on an experimental basis. According to the MD, this treatment will make a significant contribution to scientific knowledge. He does not talk about the inevitability of her death, or about the pain that will come. Vivian is fully aware that she will die. A clear example of the patient–physician interaction is shown in the ward round, a parade of doctors and students, a degrading routine with a paternalistic senior MD, who is only interested in Vivian as a research object. He addresses her with an impersonal and strictly formal politeness and plasters her with medical terms which Vivian counters with her own jargon. Doctors and technicians are by turn inappropriately cheery, overly familiar, presumptuous and rude. In the novel the divide between the patient and health care providers is literally visible (page 36): the left hand side of the page shows Vivian’s words, the right hand side of that same page shows the MD’s words – painfully showing they are not hearing each other at all, and the authoritarian demeanour of the MD.

The story of the actual patient with ovarian carcinoma about her trajectory with the condition differed substantially from the fictitious patient in *Wit*. This finding allowed discussions about the biomedical vs. the biopsychosocial model of care among the medical students, underlining the value of IPA methodology in attempting to encourage medical professionals to incorporate the patient’s story into their medical management. Not all is well though: the MD co-teacher showed a video recording of the surgical procedure in the patient in the Honours Class session. All students were totally absorbed by the video recording.

The four novels discussed here are examples of how novels represent material that can be used in health psychology research. Related articles discuss respiratory disorders, cardiovascular disease, oncology and infectious diseases as depicted in novels (Kaptein et al., 2020b; McGeechan et al., 2018). Other articles and academic books offer additional illustrations of how novels can shed light on health behaviour, illness behaviour and patient-health care provider interaction (e.g. Coles and Testa, 2002; Hunter, 1991; Oatley, 2011).

## Discussion

The material discussed in this polemical, argumentative article allows formulating two *major findings*. First, novels represent a source of data for research in health psychology. Conditions apply, however: theory-driven data collection avoids the trap of post-hoc fitting data into categories defined beforehand. Interpretative phenomenological analysis seems to be an example of such a theory-driven approach to data collection and analysis. This Journal represents a tradition of publishing articles which enlighten and support theory-driven methodologies, usually using qualitative research methods (e.g. Murray and Chamberlain, 1998). At the same time, these authors caution against ‘methodolatry’ (Chamberlain, 2000), ‘the privileging of methodological concerns over other considerations’ (p. 285) and against ‘flowcharting’ (Spicer and Chamberlain, 1996): reducing a complex subject (here: making sense of and coping with illness) to a diagram with boxes and arrows. Second, the issues of ‘methodolatry’ and ‘flowcharting’ are helpful in supporting the shift from medical humanities to health humanities: reductionist thinking and medicine-focused thinking – in medicine and in health psychology – is insulting to people making sense of and living with a chronic illness.

Additional art genres offer a context for putting the findings in *this article into perspective*. The closest to novels are graphic medicine and graphic novels, ‘the intersection of the medium of comics and the discourse of health care’ (Czerwiec et al., 2015, p. 1). The combination of (brief) text with drawings about being ill allows a quite forceful depiction of emotions in patients, which may be informative for carers, health care providers and society. Graphic Medicine also offers health psychology research many options, as demonstrated, for instance, in the article by Lo-Fo-Wong et al. (2014) in this Journal that puts the graphic novel ‘Cancer vixen’ into a Common Sense Model perspective.

This exploratory article has some *limitations*. The article does not discuss theories and methods from the literary science. Not only would that require a PhD in the area but also introductory texts on ‘literary theory’ or ‘literary science’ hardly offer any link to health psychology. Dalrymple’s somewhat sardonic advice elicits some sympathy in the author of the present article, given his experience when attending conferences on literary science: ‘ . . . on no account should junior doctors/aspiring authors consort with academics of the humanities departments of any university, for to do so was the primrose path to stylistic perdition (and they should read a great deal)’ (Dalrymple, 2007, p. 517). Another limitation pertains to the selection of themes (i.e. illness perceptions and patient–health care provider interaction). Health psychology offers much more than these themes that are feasible for the type of study performed in the current paper (e.g. sexuality, loss, depression).

Various *research and clinical implications* follow from what has been discussed herein above. The available databases that allow selecting novels on a particular topic are rather limited in scope, detail and quality. A researcher who would want to study the representation of, for instance, ovarian cancer in novels would have a difficult task finding hits within many databases. In addition, the detailing in databases of novels is usually rather limited and devoid of information about (literary or otherwise) theory and methodology. There are many implications of this finding for research: better and more extensive databases and search systems are needed. As has been become clear in the present article, the further development of theories and methodology to analyse novels is another area for additional research. The International Health Humanities Network (www.healthhumanities.org) is helpful in this regard. A strong basis of Health Humanities in academia and within medical, literary and social sciences settings is another research implication. Conferences, a Journal of Health Humanities, and Societies are additional suggestions.

Clinical applications of health humanities pertain to bibliotherapy, expressive writing and using novels in the education and training of medical students and practitioners-in-training. In bibliotherapy, a health professional (physician, clinical psychologist) prescribes the reading of novels to patients with somatic and/or psychological or psychiatric problems in order to encourage and facilitate adequate coping with these problems (McCullis, 2012). Systematic reviews and meta analyses find that consistent positive, albeit modest, effects on measures such as depression, self-efficacy, sense of belonging and finding workable solutions to the identified problems (see Kaptein et al., 2018, for reviews). The somewhat passive form of using written sources in bibliotherapy contrasts with the more active expressive writing. In this type of intervention, research summarized in systematic reviews and meta-analyses tends to report positive effects (Pennebaker, 2018). Recently, in a follow-up study of 17 years, expressive writing was even shown to predict survival in people living with HIV (Ironson et al., 2020). Explanations for these findings seem to be related to concepts such as Theory of Mind (‘an understanding that others have mental states and the process of inferring the content of these mental states’, Mar, 2011, p. 104). The Canadian research group of Mar et al. contributes to this domain specifically, for example by studying psychophysiological changes during reading of various types of literature, as reflected in MRI measurements.

This article is limited to novels as potential sources of data for health psychology research. Graphic novels represent a recent genre in the health humanities area (www.graphicmedicine.org; McMullin, 2016). Graphic medicine and art therapy are additional clinical applications in the Health Humanities field. A major medical journal invites papers on visual storytelling ‘that explore narratives of the body, health care, healing and disability’ (Nickerson, 2018, p. E368). Other art genres are waiting to be explored in more detail, in order to examine their potential contribution to health psychology and health humanities theory and clinical applications. Examples are already available: *poems* on cancer allow exploring cognitions and emotions about having an oncological condition; the coping process with the diagnosis and therapy of lung cancer is shown in the *movie* The Lake; medical students are taught about death and grief by listening to pop songs, related research explores how addiction is represented in *music* (Butler, 2009); the *painting* ‘Science and Charity’ by Picasso is a great example of how a painting can be used for teaching or research about patient-health care provider interaction. Naghshineh et al. (2008) report on the effects of teaching medical students in a museum to observe the skin of naked persons in paintings in great detail, resulting in improvements in sophistication of the diagnostic process in actual patients with dermatological problems.

Medical anthropology (Kleinman, 1988) represents an addition to the health humanities field, covering sociocultural aspects of health and illness. The important work by Fancourt (2017) is an illustration of the value of incorporating the area of art and art therapy into a health psychology framework. Critical health psychology offers a health psychology approach to the study of narratives. Its society (International Society of Critical Health Psychology, www.ischp.net ) arranges conferences, publishes papers and books on the topic (Chamberlain et al., 2018). Incorporating ‘public health’ into Health Humanities, as suggested by Saffran (2014), seems another sensible proposition (cf. Stephens, 2011).

It is somewhat ironic, given these findings, to note how in the real world of medicine and literary science, resistance is palpable and visible against Health Humanities. Resistance against Health Humanities (and one surmises, to health psychology as well), comes from various directions. Some representatives of literary theory and (medical) ethics are critical of a scientific and empirical approach to literature and its clinical applications, and towards a plea for the empirical study of the effectiveness of health humanities in teaching medical students and health-care providers (e.g. Belling, 2010). Others are fiercely opposed to narrative research and its clinical application for reasons of threats to privacy and perceived lack of professional distance in the patient-health care provider relationship (O’Mahony, 2013). Finally, those who sympathize with the cause of Health Humanities (and health psychology!) should be aware of strong implicit or explicit negative views to Health Humanities in medical students and health care providers. Medical students respond to discussions about Health Humanities by suggesting – or implicitly ridiculing – that proponents of Health Humanities are ‘romantics’. Young physicians respond by stating that they have become physicians in order to cure ill persons, not to study suffering (Shapiro et al. 2009). Empirical work on the prestige of diseases seems to support these unhelpful views: neurosurgery and cardiology are at the top (Album and Westin, 2008). It is safe to assume that Health Humanities shares the bottom position with geriatrics, dermatovenereology and psychiatry. Health psychologists involved with research and teaching in a biomedical environment are not surprised in the least, of course.
